# Exhausted through client interaction—Detached concern profiles as an emotional resource over time?

**DOI:** 10.1371/journal.pone.0216031

**Published:** 2019-05-06

**Authors:** Bettina Lampert, Christine Unterrainer, Christian Thomas Seubert

**Affiliations:** Institute of Psychology, University of Innsbruck, Innsbruck, Austria; Aga Khan University, KENYA

## Abstract

**Objective:**

To identify long-term profiles of Detached Concern (DC), based on its core dimensions detachment (D) and empathic concern (C), and to determine their association with burnout among human service professionals.

**Method:**

Self-reported data from healthcare, teaching and social professionals (N = 108) were collected in 3-waves over an 8-month period. Latent profile analysis and analysis of covariance for repeated measures were applied.

**Results:**

Five relatively stable longitudinal DC profiles emerged: (1) ‘detached’ (high D—low C; 33%); (2) ‘empathic’ (high C—moderate D; 31%); (3) ‘balanced’ (high D—high C; 21%); (4) ‘boundless’ (high C—low D, curvilinear trend; 8%); (5) ‘moderately uninvolved’ (low C—moderate D, increasing; 7%). Findings revealed profile differences based on gender (*p* < 0.05; χ^2^(4) = 9.73) and work experience (*F* [4, 103] = 3.26, *p* < .05). Differences could also be found for emotional exhaustion (*F* [4, 101] = 6.34, *p* < .001). The lowest emotional exhaustion over time occurred among balanced professionals. A stable or increasing risk of exhaustion over time was found in particular among profiles with moderate-to-low levels of detachment.

**Conclusion:**

A balanced DC protects professionals’ mental health because it is associated with the lowest levels of emotional exhaustion over time among the distinct DC profiles. Findings provide evidence-based information for education and health-promoting interventions and contribute to self-awareness of the strengths and risks of DC and burnout for human service organisations and professionals.

## Introduction

Research has focused on the issue of burnout for several decades (see for a review [1,2]), in an attempt to address the growing mental health challenges arising in the workplace. Burnout is regarded as a work-related chronic strain reaction to job stressors in which the individual suffers a persistent negative state of mind [1,3]. Emotional exhaustion refers to feelings of being emotionally overextended, a draining of energy and chronic fatigue. It reflects the core burnout dimension [4] and the central quality of burnout which is most widely reported and systematically analysed [1]. Research in the past has consistently found that emotional exhaustion is related to mental and physical health [5–8].

Burnout was first described as a prolonged response to chronic emotional and interpersonal stressors in jobs with intensive client contact, in particular for human professionals [1,9]. This focus on the provider-recipient relationships, especially the long-term emotionally involving client interaction process, was suggested as one main source of burnout particularly in early work [1]. While client interactions can be a source of emotional strain, they are also the intrinsic motivation for and fulfilment gained from working with people. It is necessary for professionals to manage their emotions at work in order to handle emotional strain with clients and maintain mental health [10]. It is especially critical for emotionally demanding and relationship intensive professions like human services, such as helping, social and teaching professions, which carry a high risk of mental health problems [4,11].

So far, however, there has been little discussion of the determinants of burnout which consider both the emotional connection and the regulation of emotions in provider-recipient relationships. In this context, theoretical and empirical evidence sees Detached Concern [12,13] as a significant process whereby human service professionals blend compassion with emotional distance in client interaction to prevent burnout [3,14–17]. Due to conceptual indistinctness in literature, a theoretically extended concept [16,18] concretizes Detached Concern (DC) as a two-dimensional strategy integrating *empathic concern* as an emotional response towards clients and *detachment* as an emotional regulator which prevents human service professionals from becoming too highly involved with their clients at work. Both components work together in a dual and dynamic process on different levels, in the context of the emotion generating and regulation process model [19,20].

Fox [12] and Lampert [18] suggested that Davis`[21] description of empathic concern is beneficial for defining the concern component of DC. Davis [21] defines empathic concern as one component of the multi-dimensional empathy concept, involving a tendency to experience feelings of warmth, compassion and concern for other people. In this respect, Lampert and Glaser [16] provided an extended definition of empathic concern by applying it to the work context and including a behavioural aspect [16,18], for example, empathically listening to clients or comforting them, since responding empathically without any behavioural reaction would be ineffective for clients [22,23]. From this perspective, Lampert and Glaser define empathic concern in the DC concept as “an other-oriented emotional response of helpers in social interaction processes at work” ([16] p. 4). It implies attuning oneself to the recipients’ emotional states and it involves experiencing feelings of warmth, compassion and concern for clients. It also involves showing a visible behavioural reaction to recipients’ emotional experiences that aims to regulate clients’ emotions, in order to carry out interaction work humanely and effectively [16]. High concern represents the anchor of a successful DC process [18], as it is a fundamental requirement for the provider-client relationship and a source of the professional’s motivation. Gleichgerrcht and Decety [24] found that empathic concern (measured with Davis`Interpersonal Reactivity Inventory [21]) was strongly related to physicians’ compassion satisfaction which describes the pleasure derived from being able to perform well on the job.

In general, empathic concern constitutes one component of the broader empathy concept [16,18,21]. The literature on empathy is diverse and there are ongoing, unresolved definitional debates concerning this concept [25]. However, most scholars agree that the concept of empathy involves both affective and cognitive elements [25]. Empathic concern in the sense of DC refers to the affective component of empathy [12,16,18] and describes an emotional response to the emotional state of another person (stimulus). Perspective taking is regarded as the cognitive component of empathy, which focuses on the understanding of another person’s emotions but not feeling them. Although there is less agreement on the definition of empathy, the literature provides substantial support that empathy is a key factor in the quality of a client relationship [24,26–34] in occupational settings. Working in an empathetic way supports the establishing of rapport with clients and helps, for example, in a medical context to gain a deeper insight into the needs of patients and their illness, while also contributing directly to therapeutic efficacy[31] . Wilkinson et al. state [30] in a systematic review that irrespective of the particular dimension or definition, empathy is related to a variety of positive patient outcomes [27,33,34] such as improved doctor-patient relationships, fewer medical errors or higher patient satisfaction.

Professionals’ empathic concern and emotional involvement are central requirements in day-to-day work with clients but might also become increasingly difficult with time when resonating emotional reactions cannot be adequately regulated whilst dealing with clients. Accordingly, detachment is seen as the necessary emotion regulation counterbalance and serves as a professional`s emotion regulation strategy to control or prevent potential emotional overload [16]. In general, Gross ([20] p. 275) defines emotion regulation as “processes by which individuals influence which emotions they have, when they have them, and how they experience and express emotions”. Lampert and Glaser describe detachment as “a helper’s emotion regulation strategy at work aiming to regulate own (helpers’) emotional states by keeping an emotional distance from the client to reduce the (negative) emotional impact.” ([16] p. 5). It is the nature of the job that clients move employees emotionally. Client`s emotions and employee`s resonating emotions can be relevant sources of information for the working process [31]. Employees need to manage these emotions in a professional way through detachment and doing so effectively is a key part of the job because it enables such employees to function in burdensome situations or deal appropriately with unfriendly clients. However, detachment is sometimes understood as a form of disengagement from clients or a way of not having to respond emotionally. We assume instead that the opposite applies as regulating own emotions (in the sense of detachment) ideally requires the individual to notice the activated emotions in themselves as a first step. Then, rather than disengaging emotionally, detachment helps a person to regulate their emotions and retain emotional distance through perceiving themselves as a separate individual in their professional role (e.g. a doctor in relation to his/her patient).

The literature on micro role transitions [35] contributes to an understanding of detachment as it discusses the process of role spillover. Ashforth et al. ([35] p. 477) describe that “mood, stress, and thoughts that are generated in one role domain often influence or spill over into other domains [36,37]”. For instance, the mother/father role in a physician might be easily called up when a young patient similar to his/her own child has to be treated. This increases the risk that burdening personal emotions are activated which can then turn into emotional distress. Bateman, White, Tofil, Clair and Needham ([38] p. 905) found in their study of end-of-life care settings with children, that those physicians who were parents in their private lives reported more emotional distress. They identified more with children in hospital which then made it more difficult for them to remain objective. Moreover, female physicians experienced more intense emotional pain with longer lingering effects arising from end-of-life interactions with children. They had more difficulty in detaching emotional pain experience during work from their lives outside of work. There should be a detachment process with rather low permeability between the professional`s work and private role, even though it might be difficult to separate these roles in certain moments. The more permeable the boundary is, the higher the risk that the professional connects too closely with the client and experiences emotional distress. It can be assumed that the boundary blurs more towards the private role and professionals become more personally involved when they identify more with clients through relational factors such as their own past experiences, personal similarities or having good interpersonal relationships with them [16]. Also, professionals with high state empathy [39] and who engage in too much compassionate responding [26] are at greater risk of suffering from another person’s emotional state. Detachment functions as an emotion regulator and reduces the risk of emotional suffering [18,40], thereby protecting an individual’s emotional resources and helping that person to maintain a professional relationship with their clients [41].

The dual perspective of the DC concept [12–14,16,18] integrates and needs both components: employee`s concern as an emotional response in the sense of connecting with clients and their needs, *and* detachment as an emotional distance regulator. The components are working together in a dynamic process at different stages in the emotion-generating and regulation process as a professional resource at work [18]. Cadge and Hammonds [14] point out that detachment and concern are negotiated in ongoing ways. According to Lampert and Unterrainer [40], the DC concept can be related to a broader qualitative approach of emotional boundary management at work [42] which is not related to a single interaction but refers to a broader approach and is part of the professional`s persona at work. This helps professionals to reach work-oriented goals and form balanced relationships with their clients. As an example in the medical context [40], a physician ideally reacts to the patient’s pain (stimulus) with empathic concern as an other-oriented emotional response This does not mean that the physician is feeling the same as the patient. Instead, it means that the physician attunes to the patient`s emotional state through feelings of warmth, compassion and concern for the patient. This process can also activate burdening emotions in the physician, in particular in situations with high involvement, which need to be handled appropriately. In a successful DC process, detachment regulates these personal emotions by differentiating between oneself in a work role and in a personal role. In doing so, also a work-private role boundary is drawn to the client. This ideally prevents the emotional state of the client crossing over to the physician or leading to emotional distress through upcoming intense experiences of emotions in the self.

**Fig 1:**
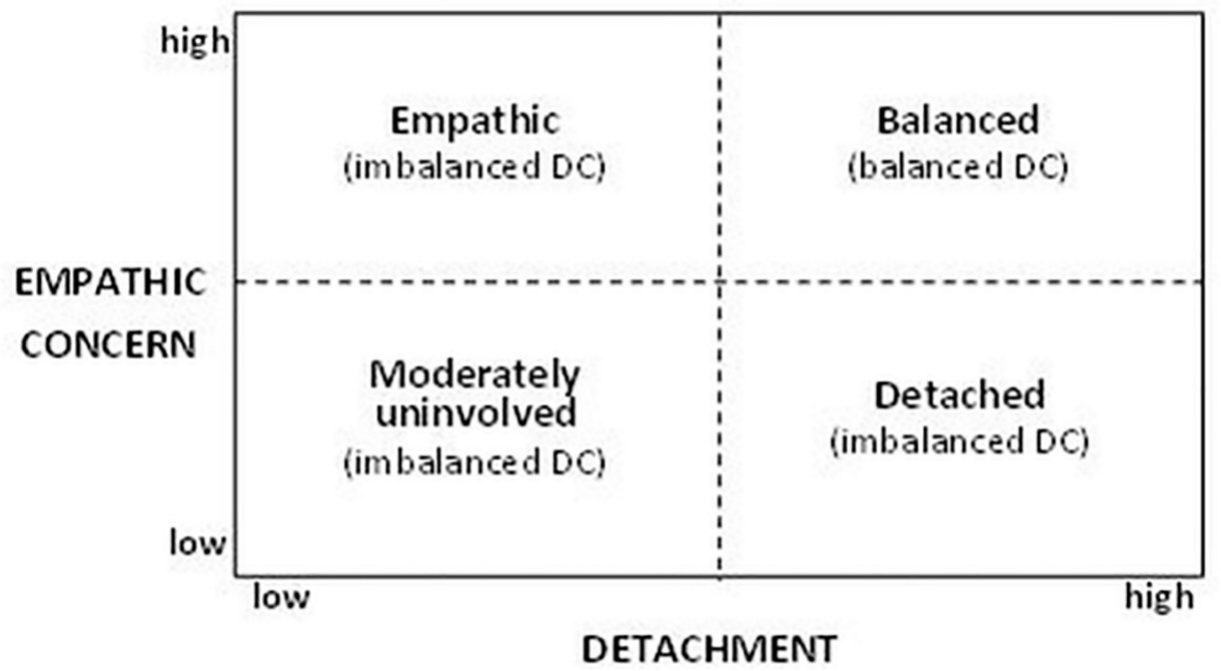
Adapted (im)-balanced DC types [40].

Quantitative studies [18,40] supported the theoretical assumption that human service providers like nurses or physicians with intense client contact manage their emotional space based on the interplay of different levels of professionals’ empathic concern and detachment at work, resulting not in one but several DC types. Four different DC types emerged in their cross-sectional studies, which are presented in Fig 1 as a simplified 2 x 2 Matrix ([40] p. 117).

Results indicated that professionals with a (highly-)balanced DC, characterized by both high empathic concern and high emotional detachment, were significantly less exhausted compared to other imbalanced professionals. Patients of balanced physicians also perceived a significantly higher care quality compared to other imbalanced types. These studies provided initial evidence for the theoretically proposed dual perspective of DC and Lief and Fox`s [13] explanation that the mixture of the two core dimensions is essential. Furthermore, Lampert and Unterrainer [40] found a higher risk of emotional exhaustion for “empathic” professionals with high empathic concern but low detachment, whereas less exhaustion was found for “detached” professionals scoring high on detachment and low on concern. The highest exhaustion risk could be found in particular for professionals scoring both moderate to low on empathic concern and detachment (“moderately uninvolved” professionals). Results of a study on DC constellations [16], analysing the nuanced interactive effects of different levels of empathic concern and detachment on burnout, found similar results. Employees’ emotional exhaustion decreased when both concern and detachment increased. The highest level of employee emotional exhaustion occurred when there was low concern and low detachment, and also when the concern level gradually exceeded the level of detachment.

These results are a starting point for providing a differentiated perspective on the impact of concern and detachment in client work on employee burnout. However, the results come from cross-sectional studies in the form of single-shot analyses rather than longitudinal approaches. Thus, several questions remain unclear which we want to address in this study. The first question is whether we can identify subgroups of human professionals with similar fluctuating, stable or even mixed levels of empathic concern and detachment over time. Taking the empathic type found in cross-sectional studies as an example: can we identify professionals applying high empathic concern and hardly any detachment over a longer period of time, in a similar and rather stable way? The identification of stable DC profiles over time could contribute to an improved understanding of the research of employees’ emotional boundary management ([42] p. 1511) as a “broader approach to work, part of a nurse`s professional persona”. Thus, the study could shed some important light on the social context of people-oriented work and the way in which professionals deal emotionally with client relationships as a principal job requirement. To the best of our knowledge, no study has yet examined the long-term interaction of the DC components. Scarce literature can be found on longitudinal empathy in the workplace context, focusing merely on healthcare settings [28,43,44] but not specifically on empathic concern. The empathic concern component can be considered to be a dispositional component in the sense of Davis [21]. Thus, it is reasonable to expect that the level of empathic concern differs between human service providers but is not volatile within a professional over time. However, in a review on medical trainees’ empathy [44], a loss of empathy was found particularly in the initial clinical practice phase of medical education. Interestingly, the issue of longitudinal emotional detachment also remains unexamined.

Due to the existing findings and outlined gaps in literature, it is reasonable to expect that DC long-term profiles imply either a relatively consistent tendency with stable levels of both detachment and concern or fluctuating DC profiles at different measurement points. Additionally, it is of utmost interest to explore if the highly-balanced DC type (integrating both high empathic concern and high detachment) found in previous studies [16,18,40] with its benefits on positive well-being and care quality can be replicated in a longitudinal design.

Closely related to this is the second question which focuses on whether such long-term DC profiles play a unique role in terms of employee burnout levels. Can professionals’ emotional exhaustion be influenced by the way professionals manage their emotional space in client interaction as time goes by? Although burnout has been found to be rather stable over longer periods of time [45], there is still a lack of longitudinal studies [46]. No research has surveyed the nature of the longitudinal relationship between the DC mechanism and burnout. A current review [30] found evidence that in eight out of ten studies cognitive and affective empathy were negatively correlated with burnout. However, in the latter review it was stated that all included studies were regrettably found to be limited to cross-sectional designs. It seems reasonable to expect that a stable high DC balance is related to a lower long-term emotional exhaustion level. Thus, it could be regarded as a sustainable and functional emotional strategy in client interaction and is worth striving for in work settings. To clarify, for whom and when balanced or imbalanced DC profiles are a preventive or risk factor for long-term well-being, could provide useful information for targeted health interventions and workplace education or care programmes.

Based on the outlined considerations, we apply an explanatory approach exploring the following research questions: Research Question (1a) What kinds of long-term DC profiles can be identified regarding the respective levels of professionals’ concern and detachment at work? (1b) Is there one long-term profile of a highly-balanced DC composed of a high level of concern and a high level of detachment? Research Question (2a) Do the different DC long-term profiles play a role in the relationship with professionals’ emotional exhaustion? (2b) Do professionals who have a highly-balanced long-term profile differ in regard to their emotional exhaustion levels compared to other profiles?

## Methods

### Data collection

The study originated from a research project funded by the University of Innsbruck which aimed to assess longitudinal profiles of participants’ concern and detachment and their effects on emotional exhaustion. As this was the first study to measure concern and detachment over time, we could not link it to previous research nor to strong theoretical assumptions for determining suitable time intervals. In this scenario, we followed the advice of Taris and Kompier [47] to include multiple waves with relatively short time intervals between the waves. Consequently, we chose to employ a short-term time lag of two months between the first two waves and a medium-term time lag of six months between the second and third wave. In summary, both paper-pencil and online surveys were distributed in three waves over a period of eight months: October 2015 (T1), December 2015 (T2; two-month time lag) and June 2016 (T3; six-month time lag). Access to the study was controlled, as only workers from a broad range of human service occupations with intense client contact were invited to participate. The participants were recruited by snowballing starting with the authors`personal networks in Austria and Germany. We also invited regional nursing homes to participate, as nurses were an under-represented occupation in our personal network but represent an important group of human service professions with intense client contact. They also served as a relevant sample in former studies on DC [14,16,18]. Interested persons could contact the project team personally via telephone or e-mail and were then informed about the study aims and procedures as well as voluntary participation, anonymity, data security and secure storage. Those persons who agreed to participate did so either verbally or via e-mail. Their consent was recorded by documenting participants’ contact details, which were subsequently used to inform them about each survey wave. Participants were also asked to spread information about the study. In summary, informed consent was obtained from the outset of the study when people made their contact information available to the project team. The survey introduction, again, included details of the study’s content and information about data security as well as information on voluntary and anonymous participation. Participants could withdraw from the study at any time.

In generating the online survey, we used the publicly available open-source online survey platform “LimeSurvey”[48]. An instance of LimeSurvey was installed on a dedicated server, hosted by the IT department of the University of Innsbruck. Survey data on the server were encrypted, and access to these data was limited to the authors. Tokens randomly generated by LimeSurvey allowed for combining the data of all three measurement points. E-mail addresses that were used to inform participants about each measurement point were separated from the survey data prior to any analyses. Participants who completed a paper-pencil questionnaire used individually created codes which could only be reconstructed by themselves. Participants who participated in the whole study were offered monetary compensation of 25 € and written feedback. In order to receive either of them, participants had to give their written informed consent and provide contact information. This information was stored separately from the survey data. Data were securely stored at the University of Innsbruck. The use of the data was strictly limited to research purposes and data were analysed anonymously.

Of 202 professionals who initially registered for participation, 192 (95.0%) contributed data for at least one measurement occasion (184 [91.1%] responded at T1, 178 [88.1%] at T2 and 115 [56.9%] at T3). After carefully screening the data to eliminate cases with too many missings, a matched longitudinal data set of *N* = 108 participants was obtained (response rate 53.5%).

### Participants

On average, the participants were 40 years old (range 23–62 years, *SD* = 10.47 years) and were mostly females (81.5%). Participants’ professions comprised teachers (29.6%), physicians (20.4%), psychotherapists/psychiatrists (19.4%), nurses and healthcare assistants (13.9%) and other professions (e.g., social education workers, physical therapists; 16.7%). The majority of participants worked full-time (56.5%). Mean work experience was 13.76 years (range 1–37 years, *SD* = 10.43 years).

### Attrition analysis

Respondents who were included in the analysis (n_i_ = 108) did not differ from participants who were excluded from the study (*n*_*e*_ = 84) in gender and working hours (full-time vs. part-time). On average, excluded participants were 4.78 years younger (*t*[180] = 3.08, *p* < .01, Cohen’s *d* = 0.47) and they had 4.35 years less work experience (*t*[180] = 2.83, *p* < .01, *d* = 0.43). Regarding the employed measures, excluded participants scored higher on empathy (*t*[182] = -2.75, p < .01, d = -0.42) than included participants. No significant group differences were found for detachment and emotional exhaustion.

### Measures

#### Detached concern (DC)

DC was assessed with the two-dimensional DC measure for human service work [16], which refers to client interaction in people-oriented work settings. Both the six-item *concern* scale (sample item: “At work, I often empathize with my clients’ circumstances in order to better understand them.”) and the five-item *detachment* scale (sample item: “I can effectively dissociate myself from my clients.”) had to be answered on a five-point intensity scale ranging from 1 (*no*, *not at all*) to 5 (*yes*, *indeed*). For concern, Cronbach’s Alpha (α) coefficients over the three measurement occasions ranged from .73 to .80 and test-retest reliability (stability) ranged from .67 to .74 (Table 1). For detachment, α coefficients ranged between .81 and .89 and stability coefficients ranged between .62 and .73.

**Table 1:** Descriptive statistics, internal consistencies, and Pearson zero-order correlations of the study variables.

Variable		*M*	*SD*	1	2	3	4	5	6	7	8	9	10	11
1	Gender (1 = male, 2 = female)	1.81	-	-										
2	Work experience (years)	13,76	10.43	-0.02	-									
3	Concern (T1)	3.97	0.56	0.33**	-0.05	(0.73)								
4	Concern (T2)	4.01	0.53	0.19	-0.14	0.74**	(0.80)							
5	Concern (T3)	3.96	0.54	0.30**	-0.11	0.67**	0.69**	(0.76)						
6	Detachment (T1)	3.81	0.67	-0.14	0.00	0.13	0.15	0.14	(0.82)					
7	Detachment (T2)	3.85	0.67	-0.02	0.05	-0.04	0.07	0.13	0.73**	(0.89)				
8	Detachment (T3)	3.90	0.65	-0.09	-0.00	0.04	0.11	0.07	0.66**	0.62**	(0.81)			
9	Emotional Exhaustion (T1)	2.78	0.85	0.16	-0.02	-0.05	-0.19	-0.11	-0.45**	-0.25**	-0.24*	(0.89)		
10	Emotional Exhaustion (T2)	2.85	0.86	0.23*	-0.03	-0.00	-0.14	-0.06	-0.46**	-0.34**	-0.36**	0.84**	(0.89)	
11	Emotional Exhaustion (T3)	2.84	0.90	0.16	-0.06	-0.00	-0.18	-0.15	-0.41**	-0.38**	-0.41**	0.74**	0.78**	(0.89)

Note. *N* = 108; matrix diagonal (in parentheses): Cronbach’s alpha; *M* = mean; *SD* = standard deviation.

* *p*<0.05

** *p*<0.01.

#### Emotional exhaustion

Emotional exhaustion was measured with the German version [49] of the revised Maslach Burnout Inventory, Human Services Survey (MBI-HSS, 3rd edition) [50]. The nine items had to be answered using a six-point frequency scale ranging from 1 (*never*) to 6 (*very often*). Cronbach’s α coefficients were .89 across all measurement occasions and stability coefficients ranged between .74 and .84 (Table 1).

#### Control variables

Gender differences were found in burnout and empathic concern. Findings indicate that women scored slightly higher on emotional exhaustion [1,51] and also reported higher empathic concern [16,24] than men. Gender has also been linked to differentiated DC patterns [16,18]. Moreover, burnout was found to be higher among younger professionals in the early years of their career [1]. Thus, gender and work experience were included as control variables.

### Statistical analysis

In order to identify possible DC long-term profiles in three repeated measurements (Research Question 1a, 1b) we conducted Latent Profile Analysis (LPA) applying maximum likelihood estimation with robust standard errors (MLR) in Mplus 8.0 [52]. LPA uses measured variables for the statistical identification of naturally occurring homogeneous latent classes of individuals that differ according to their levels of detachment and concern across the three measurement points. Since LPA is model based, we estimated different models starting from one-class solution to estimate the parameters for 2, 3, …, k-class solutions [53]. Multiple statistical indicators were used to determine the best fitting model: 1) Bayesian Information Criterion (BIC)–lowest value among competing models indicate the best fit; 2) Entropy–estimates how well the model classifies subjects with values close to 1 indicating better classification; 3) Vuong-Lo-Mendell-Rubin (VLMR); and 4) Lo-Mendell-Rubin (LMR) likelihood ratio test. VLMR and LMR compare the model fit between two nested models that vary by one class. A low *p*-value (*p* < .05) suggests that the *k* model is a significantly better fit than a *k*– 1 classes. For the decision of the most adequate class solution, we considered the meaningfulness of the classes in different class solutions, in addition to the above-mentioned statistical criteria.

In a further step, we investigated if the long-term DC profiles differed in the control variables of gender and work experience. For this purpose, we performed chi-square test (gender) and one-way analysis of variance (ANOVA) (work experience) using IBM SPSS Statistics 24. Finally, we conducted analysis of covariance (ANCOVA) for repeated measures to analyse the differences in professionals’ emotional exhaustion between the long-term DC profiles (Research Question 2a, 2b).

We refrained from employing a priori power analyses in order to determine an “optimal” sample size because (1) Tein et al. [54] found a minimal effect of sample size on the statistical power to detect the correct number of classes in LPA, and (2) power analyses concerning other variables could have been performed only after knowing the number of classes extracted by LPA, i.e., after data acquisition.

## Results

The descriptive statistics, alpha reliabilities and correlations of all variables are presented in Table 1. Participants’ mean levels of both concern and detachment were rather high, which was corroborated by the fact that 50% of all values lay in the range of 3.8 and 5 (the scale maximum level). This is an expected finding for a sample of human service professionals with intense client contact and with mid-range levels of emotional exhaustion (Table 1). Females reported higher levels of concern at T1 and T3, and higher levels of emotional exhaustion at T2. Across all measurement occasions, detachment (but not concern) related negatively to emotional exhaustion.

### Identifying long-term DC profiles

We conducted seven LPA ranging from the one-class to the seven-class solution. Table 2 reports the fit indices for the long-term DC profiles with different numbers of latent classes. Comparing the different class solutions, the fit indices supported the five-class solution most. BIC-value was lowest and the entropy value was highest in the five-class solution. The VLMR and LMR tests supported the two-class solution, but the BIC was larger and the entropy value smaller than in the five-class solution. Additionally to the information provided by the fit indices, we decided to choose the five-class solution also based on theoretical considerations. Within the five-class solution, we were able to identify four DC profiles that were already found in cross-sectional studies [40] and one new profile.

**Table 2:** Fit indices for the long-term DC profiles with different numbers of latent classes (Latent Profile Analyses).

No. of classes	Log L	BIC	Entropy	VLMR (*p*)	LMR (*p*)	Latent class proportions *n* (%)
**1**	-587.69	1231.56	-	-	-	108 (100)
**2**	-533.10	1155.16	0.82	0.006	0.007	69 (64) / 39 (36)
**3**	-492.96	1107.66	0.81	0.341	0.352	37 (34) / 28 (26) / 43 (40)
**4**	-465.71	1085.93	0.84	0.170	0.177	30 (28) / 22 (20) / 16 (15) / 40 (37)
**5**	-449.31	1085.91	0.88	0.267	0.273	9 (8) / 8 (7) / 35 (33) / 23 (21) / 33 (31)
**6**	-435.99	1092.04	0.88	0.406	0.416	9 (8) / 7 (7) / 25 (23) / 19 (18) / 13 (12) / 35 (32)
**7**	-419.29	1091.41	0.88	0.620	0.627	20 (19) / 6 (5) / 7 (6) / 14 (13) / 19 (18) / 30 (28) / 12 (11)

Note: BIC = Bayesian information criterion; VLMR = Vuong-Lo-Mendell-Rubin test; LMR = Lo-Mendell-Rubin test.

The five-class solution for the DC profiles with three measurement times is shown in Table 3. The conducted ANOVAs showed that there are several significant mean differences in detachment and concern across the five long-term DC profiles at each measurement point. Profile 1 (*n* = 35; 33%) included professionals scoring low on concern and high on detachment (= “detached” profile). Profile 2 (*n* = 33; 31%) represented participants with high scores on concern and moderate scores on detachment (= “empathic” profile). Profile 3 (*n* = 23; 21%) displayed participants reporting high concern and high detachment (= “balanced” profile) at all three measurement points. Profile 4 (*n* = 9; 8%) comprehended professionals with high concern and low detachment (= “boundless” profile). Profile 5 (*n* = 8; 7%) represented professionals with low concern and moderate detachment (= “moderately uninvolved” profile).

**Table 3:** The five-class solution for the DC profiles with three measurement times (*N* = 108) (Latent Profile Analyses). ANOVA.

DC patterns	*n* (%)	Concern			Detachment		
		T1 *M* (*SE*)	T2 *M* (*SE*)	T3 *M* (*SE*)	T1 *M* (*SE*)	T2 *M* (*SE*)	T3 *M* (*SE*)
**1. Low concern, high detachment (Detached)**	35 (33)	3.59 (0.05)	3.62 (0.06)	3.58 (0.06)	4.13 (0.06)	4.09 (0.05)	4.10 (0.08)
**2. High concern, moderate detachment (Empathic)**	33 (31)	4.22 (0.08)	4.26 (0.06)	4.19 (0.07)	3.52 (0.07)	3.55 (0.07)	3.67 (0.08)
**3. High concern, high detachment (Balanced)**	23 (21)	4.34 (0.08)	4.43 (0.08)	4.47 (0.06)	4.44 (0.08)	4.58 (0.07)	4.44 (0.10)
**4. High concern, low detachment (curvilinear trend) (Boundless)**	9 (8)	4.33 (0.13)	4.24 (0.13)	3.96 (0.17)	2.82 (0.16)	2.44 (0.10)	2.91 (0.25)
**5. Low concern, moderate detachment (increasing) (Moderately uninvolved)**	8 (7)	3.13 (0.16)	3.17 (0.08)	3.29 (0.17)	2.90 (0.16)	3.44 (0.12)	3.53 (0.16)
***F*-test**		26.70***	34.48***	27.57***	48.07***	80.09***	20.24***
**Pairwise comparisons^1^**		1 < 2,3,4 5 < 1,2,3,4	1 < 2,3,4 5 < 1,2,3,4	1 < 2,3 3 > 4 5 < 2,3,4	1 > 2,4,5 2 > 4,5 3 > 1,2,4,5	1 > 2,4,5 2 > 4 3 > 1,2,4,5 4 < 5	1 > 2,4,5 2 > 4 3 > 2,4,5

Note

***p<0.001

^1^Significant Bonferroni comparisons.

Overall, the empathic concern and detachment levels were relatively stable across all profiles over 8 months. However, mean levels suggested a slight variation for concern and detachment in profile 4 (= boundless) and for detachment in profile 5 (= moderately uninvolved). The mean values for profile 4 (= boundless) indicated a slight decrease in concern and a curvilinear variation in detachment. These effects lacked statistical significance. The mean values for profile 5 (= moderately uninvolved) referred to an increase in detachment (*p* = .02). However, the pairwise comparisons indicated a statistical tendency only between T1 and T3 (*p* = .09).

### Five long-term DC profiles and differences in control variables

The DC profiles differed significantly in terms of gender (χ^2^(4) = 9.73, *p* < .05). The majority of men in the sample were found in the detached pattern (60%). Here, the male professionals were exceedingly over-represented compared to the percentage of women in the sample (26.1%). The highest proportion of women (34.1%) were found in profile 2 (= empathic) compared to 15.9% of men. The conducted ANOVA also revealed a significant difference between the DC profiles in employees’ work experience (*F* [4, 103] = 3.26, *p* < .05). Post-hoc tests (Games-Howell) showed that employees in profile 1 (= detached) worked significantly longer (*M* = 16.01 years) in their profession than employees in profile 2 (= empathic) (*M* = 8.58 years). We return to these results in the Discussion section.

### Five long-term DC profiles related to emotional exhaustion

Table 4 presents the means and mean changes of emotional exhaustion in the five long-term DC profiles. The performed analysis of covariance for repeated measures showed a significant 5 (profile) x 3 (time) interaction effect (*F* [8, 202] = 2.60, *p* < .05) and significant profile differences (*F* [4, 101] = 6.34, *p* < .001) for emotional exhaustion. Emotional exhaustion was stable over time in profile 1 (= detached), 3 (= balanced) and 5 (= moderately uninvolved). Emotional exhaustion increased in profile 2 (= empathic) from T1 to T2 and decreased again at T3. In profile 4 (= boundless) emotional exhaustion was stable from T1 to T2 and increased at T3.

**Table 4:** Changes in emotional exhaustion according to five long-term DC profiles, using analysis of covariance (ANCOVA) for repeated measures. (Gender and work experience were controlled for).

Emotional Exhaustion	1. Low concern, high Detachment (Detached)	2. High concern, moderate Detachment (Empathic)	3. High concern, high Detachment (Balanced)	4. High concern, low Detachment (curvilinear trend) (Boundless)	5. Low concern, moderate Detachment (increasing) (Moderately uninvolved)
	(*n* = 35)	(*n* = 33)	(*n* = 23)	(*n* = 9)	(*n* = 8)
	*M* (*SE*)	*M (SE)*	*M* (*SE*)	*M* (*SE*)	*M* (*SE*)
**T1**	2.62 (0.14)	2.97 (0.15)	2.40 (0.17)	2.97 (0.27)	3.59 (0.29)
**T2**	2.64 (0.13)	3.24 (0.14)	2.32 (0.16)	2.98 (0.26)	3.54 (0.27)
**T3**	2.71 (0.14)	2.98 (0.15)	2.33 (0.17)	3.55 (0.28)	3.59 (0.29)
**Pattern differences**		*F*		6.34***	
**Time effect**		*F*		0.96 ns	
**Pattern x Time**		*F*		2.60*	

Note:

*p<0.05

**p<0.01

***p<0.001

**Fig 2:**
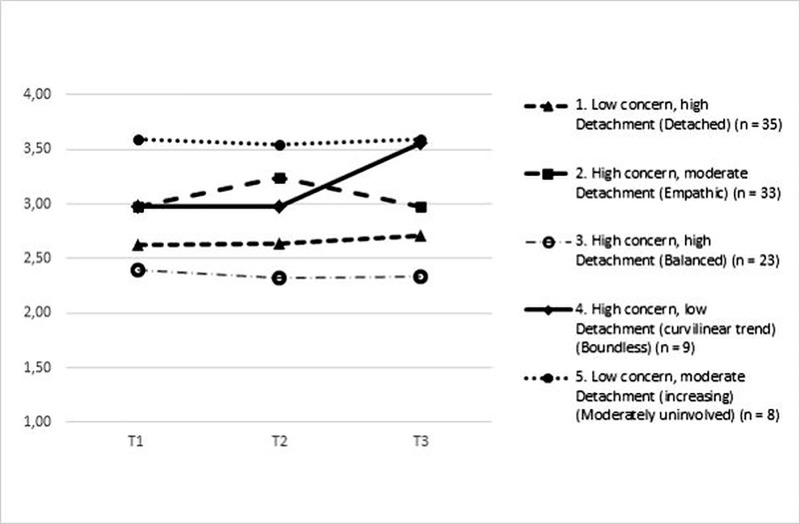
Emotional exhaustion at each time point in the five long-term DC profiles.

The mean differences between profiles at each time point are displayed in Fig 2 (Bonferroni comparisons). Profile 1 (= detached) and 3 (= balanced) reached significantly lower values in emotional exhaustion than profile 5 (= moderately uninvolved) at T1 and T2. Profile 1 (= detached) and 3 (= balanced) also scored significantly lower on emotional exhaustion than profile 2 (= empathic) at T2. At T3, emotional exhaustion was significantly lower in profile 3 (= balanced) compared to profile 4 (= boundless) and 5 (= moderately uninvolved). At all three measurement points, the balanced profile (high detachment and high concern) was the healthiest with the lowest levels of emotional exhaustion.

## Discussion

The aim of the present study was to focus on extending our understanding of how and why professionals regulate their emotional space in client-interactions as part of a required emotional involvement process in human service jobs. More specifically, the first goal was to explore potential person-centered profiles of professionals’ empathic concern and detachment as an integral part of the DC concept and its longitudinal development. The second aim was to extend the knowledge of cross-sectional findings that pertain to the relationship between long-term DC profiles and burnout among human service professionals. We investigated how yet unidentified long-term DC profiles of empathic concern and detachment developed in an 8-months follow-up study using 3 waves. Human service professionals formed the target group, as their work is regarded as emotionally demanding and involves the provision of empathic face-to-face services with intensive client contact, as a principal work task.

The major findings of our study provided the first longitudinal support for the view that there is not one DC but several distinct DC profiles depending on the interplay of various combinations of concern and detachment on the part of a professional. We identified five different long-term profiles. Four out of five of these DC profiles were consistent with previous cross-sectional findings [18,40] but gave a deeper understanding of DC by adding a longitudinal quality to it: (a) “detached” profile 1 (low levels of concern and high levels of detachment at all time points), (b) “empathic” profile 2 (high levels of concern and moderate levels of detachment at all time points), (c) “balanced” profile 3 (high concern and high detachment at all time points), and (d) “moderately uninvolved” profile 5 (low levels of concern at all time points and moderate detachment at baseline with a slight increase over time). Also, a new profile emerged of professionals (“boundless” profile 4) who reported high concern and low detachment over time. The mean values showed modest variations over time but lacked statistical significance. The boundless professionals scored the lowest detachment at all time points compared to the other profiles.

Overall, these findings are novel as they establish that professionals have distinct but relatively stable ways of responding empathically and also detach from their clients as form of emotional regulation, which protects them over time. Professionals in different profiles scored fairly consistent results with similar DC levels of higher or lower empathic concern or detachment over 8 months. The DC profiles can be seen as part of developing a professional identity [13] in relation to the client interaction process at work which affects how professionals balance their concern and detachment [55].

Importantly, of the five profiles, the highly balanced DC turned out to be the healthiest profile when it comes to the differences between those with higher and lower burnout levels. We could demonstrate that professionals belonging to the highly balanced profile with persistently high levels of concern and detachment over time perceived no suffering from emotional strain at any point over the eight months. This is in line with previous cross-sectional findings and highlights a potential longitudinal prevention function of a highly-balanced successful DC. Notably, of the two DC dimensions, detachment turned out to be an important determinant for emotional exhaustion. We found (Table 1) that detachment, rather than empathic concern, showed significant moderate to strong negative correlations with emotional exhaustion on all measurement points. This result is also in line with previous studies [16], where detachment had the strongest negative effect on emotional exhaustion. This underlines that the regulation of own emotions is a key element of personal well-being [56], as the main purpose of detachment is an intrapersonal regulation process of emotions in which the professionals retain a personal boundary between them and their clients. In other words, high detachment functions as a powerful protector against emotional exhaustion and helps the professionals to maintain their emotional resources. Also, within profile 1 (= detached) the second highest constant detachment levels could be found to exceed the low concern levels on all three time points. The exhaustion risk of the detached professionals was permanently low over the 8 months. However, caution is advised in interpreting this result. This is because it is important to note that even though detachment can prevent emotional exhaustion, this should not lead to a misunderstanding whereby one emphasizes detachment over concern [14]. Concern is a powerful indicator for a successful and healthy DC balancing process that shapes interaction between professionals and clients. It also affects positive outcomes such as patient-centeredness, patient satisfaction as well as job motivation on the employees’ side. This point is discussed later.

All profiles, characterized by a moderate to low detachment level (boundless, empathic or moderately uninvolved professionals), were at higher risk of experiencing emotional exhaustion over time. In particular, moderately uninvolved professionals were vulnerable to experiencing persistent emotional exhaustion over the 8 months, feeling on average more exhausted rather than less exhausted at each measurement point. This profile had the smallest group size of 8 professionals (7.4%). According to Bakker and Costa [6], professionals with high chronic burnout levels are more likely to experience a loss cycle of exhaustion and fail to benefit from a gain cycle of accumulating resources [57] because they lack the energy needed to do so. Once emotional exhaustion is experienced it seems to be difficult to recover, as burnout is considered to be a relatively chronic condition with stability coefficients of .50 to .70 across periods from 1 up to 10 years [45]. A similar result could also be confirmed in our study with a stability coefficient of .74 over 8 months.

Another notable finding of the study was that boundless professionals showed a distinct tendency to perceive emotional exhaustion levels that were as high as the moderately uninvolved professionals, in particular at time point three compared to 6 months earlier. All in all, they showed the lowest detachment of all profiles combined with high concern at all time points. It seems that they effectively ‘fuse’ together with their clients maintaining hardly any emotional boundaries with their clients. The boundless professionals’ strong lack of detachment over time might be a key determinant in increasing their emotional exhaustion. This could lead to the circumstance that boundless professionals might also be caught in a loss cycle [6,57] of exhaustion as time goes on and, in turn, they increasingly lack the energy for detachment as an effortful emotion regulation process.

A closer look at the related profile of empathic professionals outlined high concern scores persistently outmatching the moderate detachment scores over 8 months, with the latter higher than the scores for boundless or moderately uninvolved professionals. Emotional exhaustion levels increased after 2 months and settled after another six month. Exhaustion was in general lower for empathic compared to moderately uninvolved professionals. Exhaustion was also partially lower for empathic compared to boundless professionals. However, we identified a higher exhaustion risk at all time points for empathic professionals than for the balanced and detached professionals. So why do professionals highly engage in empathic concern but do not sufficiently apply detachment when it exhausts them? Self-determination theory [58] could be a relevant motivational approach to explain that boundless and empathic professionals might have a strong need for relatedness. In this context, Niven describes compassion motives ([59] p. 311), with a focus on helping others that originates from feelings of empathy. The driving motivation is to benefit others in the sense of prosocial motivation [60], pleasure motives [61] or philantropic emotion management [62], even though the individual costs such as exhaustion can be high [63]. This is particularly the case for boundless professionals. Even though the personal costs for such professionals are higher due to low detachment and the risk of identifying too closely with patients, the other side of the coin is that high empathic concern benefits their clients and contributes to their motivation. This may be because they find such an approach to be personally gratifying. The motivation of caring is associated with positive feelings [64] which reinforce the behaviour of caring for others.

We also found gender effects. Empathic concern was higher for women than for men. This result is not surprising, as in terms of gender, evidence confirms male–female differences in self-reported empathic concern [16,24,65] and in empathy in general [66] such that women display higher values than men, possibly due to evolutionary processes [24,64]. This result is also in line with the finding that the empathic profile included more than double the number of female professionals compared with male professionals. The opposite applied to the detached profile which included many more men than women. These results were also found in a study on nurses [18]. It is of particular interest to note that the empathic professionals’ work experience was on average 8.5 years (with 42% having 4 years maximum work experience), whereas professionals in all remaining profiles had on average double the amount of work experience ranging from M = 15.4 years (balanced professionals) to M = 17.3 years (moderately uninvolved professionals). In particular, young professionals might start with a highly empathetic working style in client interactions and low detachment at the beginning of their career. This might change with increased work experience, as a process of work adjustment and gaining knowledge and skills on the job. Empathy is a rather stable component but findings in medical students also demonstrated that it underlies changes, in the first years in particular [28,43,44] for several reasons. For example, increased patient contact and distress were reasons for a decline in empathy during the clinical practice phase of training [44] but this could also be put down to organisational factors, such as an increased use of technology, economic pressure or time constraints [33]. Preventing a decline in empathy, particularly in the early years of a professional career, should also be a focus for training [33].

In the context of emotional boundary management, Hayward and Tuckey [42] also discuss a personal development process for nurses. With experience, training and increased competence, nurses’ use of antecedent-focused emotion regulation strategies may develop into cognitive networks and contribute to ‘mental representations of their role as a nurse’ ([42] p. 1517) and form part of their professional identity. This underlines DC as an aspect of a professional’s role and his/her work identity development in becoming a nurse or psychotherapist, for example. From this development process, values and attitudes also emerge in connection with the work role [13]. Based on our results for empathic or boundless employees, it is more constructive to continue (and not reduce) working in a sympathetic way by responding to clients’ needs whilst increasing detachment at work in a balanced manner. This supports both interaction parties–the professionals and their clients. However, due to some qualitative participant feedback during health interventions on DC, conducted by the first author, professionals’ notions of increasing detachment is sometimes mixed with thoughts of being insensitive, disengage or turning away from the client and his/her needs. This is often associated with emotions of guilt that can interfere with effective emotion regulation. It is therefore critical to provide concrete information on what detachment is. It supports a process of caring for one self which is “necessary in order to have the energy to compassionately care for others” ([67] p. 613), especially while working in emotionally challenging settings where clients are in need and vulnerable [68].

### Limitations and implications for future research

Despite our study having several strengths, we are aware of limitations when interpreting our findings. First, we refrained from employing a priori power analyses (see Methods section for a justification). However, we employed retrospectively both an a priori and a post-hoc power analysis for group differences regarding emotional exhaustion (the main outcome of this study) using G*Power [69]. Regarding the a priori power analysis to determine an optimal sample size for the detection of mean differences of five DC profiles in emotional exhaustion (Table 4) at an α error probability of .05, a statistical power of .80, and an effect size *f* = 0.33 (derived from Lampert & Unterrainer [40]), a total sample size of 95 was suggested. In terms of the post-hoc power analysis used to determine the achieved statistical power for detecting the mean differences of five DC profiles in emotional exhaustion (Table 4), we used an α error probability of .05, a sample size of 108, and an obtained effect size of *f* = 0.50, resulting in a statistical power of >.99. Therefore, although we employed power analyses only after data acquisition, the results suggest that this study was based on an adequate sample size and high statistical power to detect group differences.

A further limitation pertains to the measures. Both constructs, DC and emotional exhaustion, were self-rated at all three time points, which may have induced common method bias [70]. However, since DC and emotional exhaustion are psychological constructs that measure personal states of individuals, we assume that the surveyed employees gave answers which were more closely aligned with their reality than would emerge from supervisor ratings or from objective observations. A second potential limitation concerns the time lags used for our study. Due to our boundless professionals’ findings, whose emotional exhaustion increased after 8 months to the same high level of the moderately uninvolved, the question arises: are boundless professionals a precursor to moderately uninvolved professionals in the long-run? However, the further development of emotional exhaustion in this group over a longer time interval remains unclear. It would be worth observing these processes over longer periods of time and also consider factors that influence a DC process. In this context, it seems relevant to note that emotional exhaustion was negatively related to detachment over time. In order to properly understand the relationship between DC and burnout, reversed causal relationships between those constructs across time need to be considered. This is a matter which is open to future research. Also, being exposed to a stressful working environment (e.g. workload, patient demands) or a supportive working environment (e.g. autonomy at work, social support) influences the client interaction process and may hinder or foster a DC balance. Employees probably react to such environments, e.g. with decreasing or increasing concern and detachment. Future research may overcome this shortcoming in exploring DC antecedents.

Moreover, the question to what extent DC underlies a developmental process in different stages of a career needs to be explored in depth, especially the post hoc explanation that empathic professionals are probably acquiring their work identity in a gradual way through client interactions. A qualitative approach might be a valuable alternative way of exploring this question.

Another limitation is that we investigated the impact of the five long term DC profiles exclusively on emotional exhaustion as the core burnout dimension. Future studies could examine how the two other burnout dimensions–depersonalisation and personal accomplishment–as well as other mental and physical health outcomes, e.g. psychosomatic complaints, are related to the long-term DC profiles. Additionally, as mentioned above, another promising avenue of research may investigate the impact of the DC profiles not only on health outcomes but also on employees’ motivational components, like work motivation and engagement.

### Practical implications

The beneficial and detrimental long-term effects of distinct DC profiles and burnout reveal important evidence-based information for interventions. Through this, professionals gain a differentiated understanding that the balanced interplay between both DC components–care for themselves through detachment on the one hand and being emotionally attuned to their clients, whilst keeping their motivation up through concern on the other–is highly relevant. To provide this information would be a valuable step in developing self-awareness, e.g. in a health circle, and providing a better understanding of the individual DC profiles and their effects on mental exhaustion. It could be a starting point for participants to reflect on their emotional boundary management and personal resources, helping them to steer their DC into a balanced direction, if they so wish. Techniques that focus on emotional self-awareness and self-reflection, such as mindfulness or Balint groups, are key elements which support empathy as they place a focus on clients’ and professionals`own emotions rather than avoiding or neglecting them. These emotion-centred techniques can also be used to reflect on both DC components to find the right DC balance. Different intervention approaches for the distinctive DC profiles could be most effective in alleviating burnout. In particular, high empathic professionals with low detachment are at high risk of experiencing emotional exhaustion, whereas detached professionals might miss feelings of personal accomplishment at work and the motivational aspects of fulfilling provider-client relationships. Thus, high empathic professionals who want to achieve a more balanced DC type may benefit more from training which focuses on detachment, whereas professionals with high detachment and low concern might profit more from interventions focusing on empathic concern. As mentioned above, gender and work experience should also be considered in interventions. Becoming a balanced professional might also include a challenging adaptation process to find the right dose between empathic concern for the client and detachment for oneself, resulting in a balanced DC.

Moreover, contextual factors should also be taken into consideration in creating an environment that supports DC. Several scholars [12,14,18,16,32] point out the institutional environment and cultural norms as relevant factors which are likely to influence the development of empathy. Organisational factors such as heavy workload, increased use of technology or economic pressure are likely to affect the ability of professionals to empathise with clients [12,14,26,18,32,33] or to find a DC balance. For example, a study in nursing homes for the elderly showed favourable conditions for professionals’ autonomy to be positively related to empathic concern, whereas workload showed a negative relationship with detachment [18]. Furthermore, a culture of detachment is not conductive to the development of empathy at work [32]. Even though the importance of empathy is emphasised in healthcare and medical settings, a cultural norm of maintaining objective professionalism by remaining detached from emotions can also be observed as a dominant theme in education and training programs for human service professionals, especially in the field of medicine [31,32]. When appropriate working conditions and a cultivation of empathy are actively promoted in organisations [32], professionals are given more freedom to attend courses dealing with emotional self-awareness and self-reflection and then integrate it into their day-to-day work with clients. In turn, this supports a DC balance and contributes to functional client interactions. The differential findings of this study provide relevant evidence to institutions that it is worth integrating both DC components at an early stage of a health care professional’s education, given that a balanced DC is connected with lower emotional exhaustion in the long-run. Leaders or supervisors, in particular, should be sensitised to their own DC balance and to the influences of their organisational environment on DC. They are role models to their staff and have the power to shape contextual factors such as shared values or the level of autonomy in the workplace.

## Conclusion

Our focus in this study has been on the ways that professionals with intense client contact at work manage emotional boundaries in client interactions in the long-run, and how this affects their emotional exhaustion. As the study demonstrates, employees obviously work in a distinct way with clients applying more or less empathic concern in combination with more or less emotion regulation through detachment, resulting in five different DC profiles. Over a longer period of time employees seem to favour one of these DC profiles as part of their professional identity. Gender and work experience also interfere with the DC profiles. Professionals are more prone to experience emotional exhaustion when they emphasize one of the DC dimensions (detachment- empathic concern) over the other. A highly-balanced successful DC is most beneficial for the prevention of burnout and complements a positive approach to emotions and emotion regulation in provider-client interaction.

## Supporting information

S1 File Data file with anonymised longitudinal data. (SAV)
